# The Raf kinase inhibitors Dabrafenib and Regorafenib impair Zika virus replication via distinct mechanisms

**DOI:** 10.1128/jvi.00618-24

**Published:** 2024-07-18

**Authors:** Lucas Wilken, Guus F. Rimmelzwaan, Husni Elbahesh

**Affiliations:** 1 Research Center for Emerging Infections and Zoonoses (RIZ), University of Veterinary Medicine (TiHo), Hannover, Germany; University of Kentucky College of Medicine, Lexington, Kentucky, USA

**Keywords:** flavivirus, kinase inhibitors, RNA virus, host-directed antivirals, drug repurposing

## Abstract

Zika virus (ZIKV) is a re-emerging mosquito-borne flavivirus that has been associated with congenital neurological defects in fetuses born to infected mothers. At present, no vaccine or antiviral therapy is available to combat this devastating disease. Repurposing drugs that target essential host factors exploited by viruses is an attractive therapeutic approach. Here, we screened a panel of clinically approved small-molecule kinase inhibitors for their antiviral effects against a clinical isolate of ZIKV and thoroughly characterized their mechanisms of action. We found that the Raf kinase inhibitors Dabrafenib and Regorafenib potently impair the replication of ZIKV, but not that of its close relative dengue virus. Time-of-addition experiments showed that both inhibitors target ZIKV infection at post-entry steps. We found that Dabrafenib, but not Regorafenib, interfered with ZIKV genome replication by impairing both negative- and positive-strand RNA synthesis. Regorafenib, on the other hand, altered steady-state viral protein levels, viral egress, and blocked NS1 secretion. We also observed Regorafenib-induced ER fragmentation in ZIKV-infected cells, which might contribute to its antiviral effects. Because these inhibitors target different steps of the ZIKV infection cycle, their use in combination therapy may amplify their antiviral effects which could be further explored for future therapeutic strategies against ZIKV and possibly other flaviviruses.

**IMPORTANCE:**

There is an urgent need to develop effective therapeutics against re-emerging arboviruses associated with neurological disorders like Zika virus (ZIKV). We identified two FDA-approved kinase inhibitors, Dabrafenib and Regorafenib, as potent inhibitors of contemporary ZIKV strains at distinct stages of infection despite overlapping host targets. Both inhibitors reduced viral titers by ~1 to 2 log_10_ (~10-fold to 100-fold) with minimal cytotoxicity. Furthermore, we show that Dabrafenib inhibits ZIKV RNA replication whereas Regorafenib inhibits ZIKV translation and egress. Regorafenib has the added benefit of limiting NS1 secretion, which contributes to the pathogenesis and disease progression of several flaviviruses. Because these inhibitors affect distinct post-entry steps of ZIKV infection, their therapeutic potential may be amplified by combination therapy and likely does not require prophylactic administration. This study provides further insight into ZIKV-host interactions and has implications for the development of novel antivirals against ZIKV and possibly other flaviviruses.

## INTRODUCTION

The endemicity of many arboviruses, including Zika virus (ZIKV) and dengue virus (DENV), in many low- and middle-income countries means that nearly half of the global population lives in areas at risk of zoonotic arbovirus infections (1). ZIKV is a re-emerging virus that is transmitted to humans primarily by infected mosquitoes (*Aedes aegypti* and *Aedes albopictus*). There are two ZIKV lineages, African and Asian, the latter of which was responsible for smaller outbreaks in Micronesia and French Polynesia and more recently the 2015–2016 outbreak in the Americas which spread to more than 80 countries (2). However, climate change has driven habitat expansion of competent vectors like *Ae. aegypti* and *Ae. albopictus* increasing the risk of ZIKV transmission beyond their endemic habitats (3). Although the vast majority of ZIKV infections are mildly symptomatic or asymptomatic in immunocompetent patients (4), they can be catastrophic for pregnant women resulting in spontaneous miscarriages, still-births, and several congenital abnormalities including microcephaly (5–7). Additionally, ZIKV infections have an increased risk of neurological complications including Guillain–Barré syndrome, encephalitis, and/or myelitis in young children and adults (8–11). Despite potentially catastrophic outcomes, no vaccine or antiviral treatments are available.

ZIKV is a member of the genus *Flavivirus*, which also includes other important human pathogens including the four serotypes of DENV (DENV1–4), West Nile virus (WNV), Japanese encephalitis virus (JEV), and yellow fever virus (YFV). The flavivirus genome is composed of a positive-sense, single-stranded RNA that after viral entry into host cells is immediately translated at the rough endoplasmic reticulum (ER) into a single polyprotein that is co- and post-translationally processed into three structural proteins [capsid, precursor membrane (prM), and envelope (E)] and seven non-structural proteins (NS1, NS2A, NS2B, NS3, NS4A, NS4B, and NS5). The non-structural proteins induce massive remodeling of ER membranes, resulting in the formation of viral replication organelles. These are predominantly composed of arrays of invaginated vesicles, referred to as vesicle packets, in which viral genome replication is thought to take place (12, 13). Viral RNA amplification occurs through a negative-sense antigenome, leading to formation of double-stranded RNA (dsRNA) replication intermediates, and depends on the enzymatic activities of NS5 (RNA-dependent RNA polymerase and methyltransferase) and NS3 (helicase and nucleoside triphosphatase) (14). The resulting positive-sense RNA is then used for subsequent translation or formation of progeny virus. Virion assembly occurs in close proximity to the sites of viral genome replication, often directly juxtaposed to the vesicle packets (13, 15). Newly synthesized viral RNA associates with capsid protein to form a nucleocapsid, which then buds into the ER lumen to acquire a lipid envelope harboring the prM and E proteins. Non-infectious, immature (prM protein-containing) virions are transported along the secretory pathway to the extracellular space. Maturation occurs in the *trans*-Golgi network (TGN), where cleavage of prM protein by furin or furin-like proteases renders flavivirus particles infectious (16).

Antiviral treatment strategies have typically focused on direct targeting of viral proteins, like proteases and polymerases. This often results in the rapid emergence of drug resistance, especially among RNA viruses, which usually have high mutation rates, and consequently renders such drugs ineffective (17). In contrast, targeting host factors required for viral replication poses a higher genetic barrier to resistance due to the considerable number of adaptive mutations needed to use alternative host factors (18). Ideally, the substantial overlap between host factors and pathways required by different viruses could be exploited to allow for broad-spectrum use of these drugs (19).

A growing number of small-molecule kinase inhibitors (SMKIs) have gained approval by the Food and Drug Administration (FDA) as treatment options for multiple types of cancer as well as autoimmune and inflammatory disorders (20). The two major types of kinases are serine/threonine kinases and tyrosine kinases (receptor and non-receptor) which are essential components of signal transduction pathways, regulate key cellular processes exploited by viruses, and have been shown to phosphorylate individual viral proteins (21). Some kinases have already been implicated in the ZIKV replication cycle. For instance, the receptor tyrosine kinase AXL serves as a cofactor for viral entry into glial cells (22, 23) and umbilical vein endothelial cells (24); and Src family kinases have been shown to be important for viral egress (25). In this study, we examined various FDA-approved SMKIs as potential host-directed antivirals against ZIKV.

## RESULTS

### Dabrafenib and Regorafenib inhibit ZIKV but not DENV2 replication

**Fig 1:**
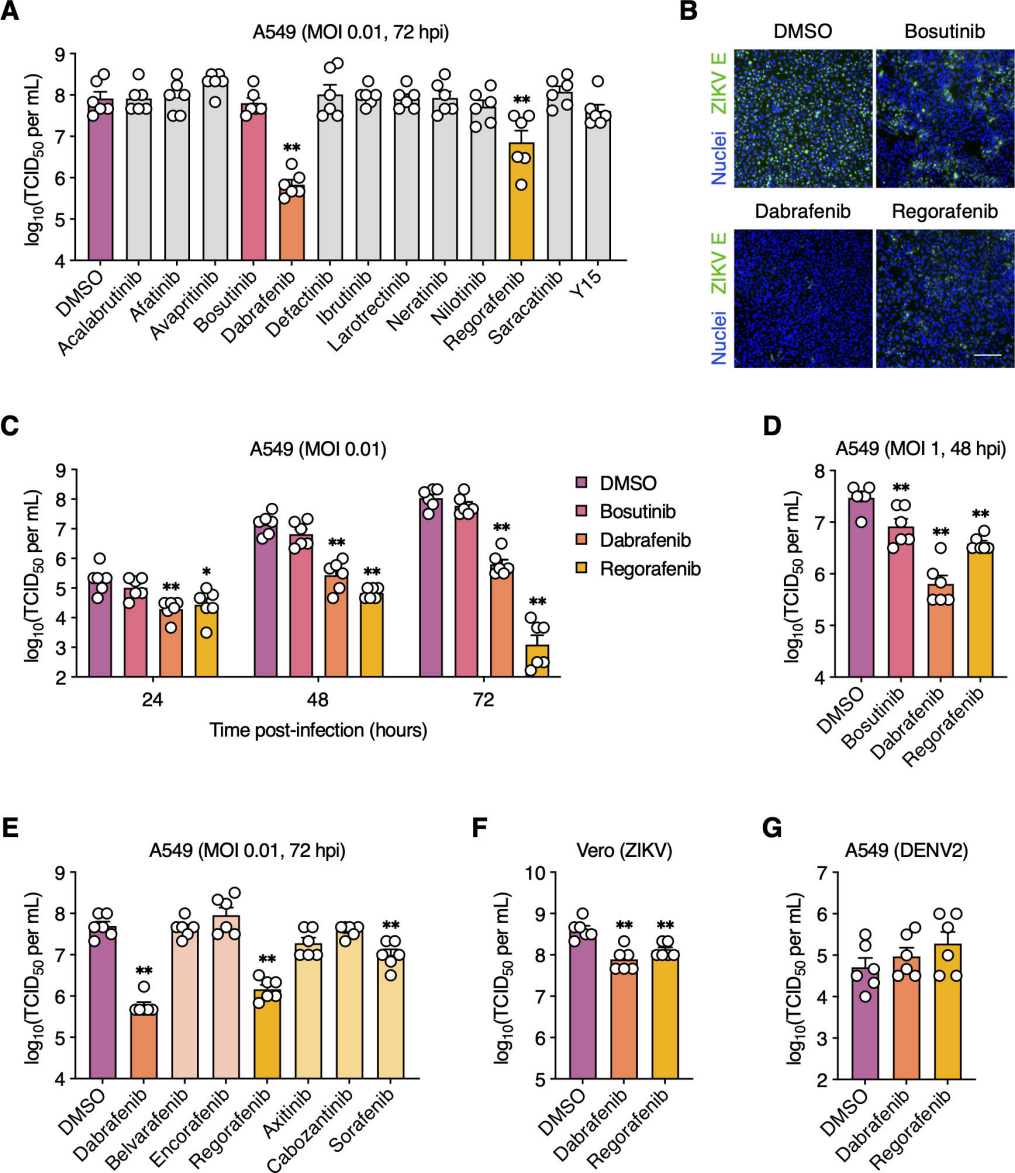
Dabrafenib and Regorafenib inhibit ZIKV but not DENV2 replication. (**A and B**) A549 cells were mock-infected or infected with ZIKV (MOI = 0.01 PFU/cell) and subsequently treated with the indicated SMKIs or DMSO (vehicle control). (**A**) Viral titers in the supernatant were determined at 72 hpi by endpoint dilution assay. (**B**) ZIKV infection was visualized at 72 hpi by immunofluorescence staining for ZIKV E protein (green). Nuclei were stained with Hoechst 33342 (blue). Scale bar, 200 µm. Representative images of two independent experiments are shown. (**C**) A549 cells were infected with ZIKV (MOI = 0.01 PFU/cell) and subsequently treated with DMSO, bosutinib (5 µM), Dabrafenib (10 µM), or Regorafenib (2.5 µM). Viral titers in the supernatant were determined at 2, 24, 48, and 72 hpi by endpoint dilution assay. (**D**) A549 cells were infected with ZIKV (MOI = 1 PFU/cell) and subsequently treated with DMSO, bosutinib (5 µM), Dabrafenib (10 µM), or Regorafenib (2.5 µM). Viral titers in the supernatant were determined at 48 hpi by endpoint dilution assay. (**E**) A549 cells were infected with ZIKV (MOI = 0.01 PFU/cell) and subsequently treated with the indicated SMKIs or DMSO. Viral titers in the supernatant were determined at 72 hpi by endpoint dilution assay. (**F**) Vero cells were infected with ZIKV (MOI = 0.01 PFU/cell) and subsequently treated with DMSO, Dabrafenib (2.5 µM), or Regorafenib (1 µM). Viral titers in the supernatant were determined at 48 hpi by endpoint dilution assay. (**G**) A549 cells were mock-infected or infected with DENV2 (MOI = 0.01 PFU/cell) and subsequently treated with DMSO, Dabrafenib (10 µM), or Regorafenib (2.5 µM). Viral titers in the supernatant were determined at 72 hpi by endpoint dilution assay. Data are expressed as mean ± standard error of the mean (SEM) from two independent experiments with three biological replicates per experiment. *, *P* < 0.05; **, *P* < 0.01 (Mann–Whitney *U* test vs “DMSO”).

We initially screened 13 FDA-approved SMKIs, which we previously identified as potent inhibitors of influenza A virus replication *in vitro* (26–28), for their antiviral activity against a low-passage clinical isolate of ZIKV (strain FB-GWUH-2016) (29). As a benchmark, we included Bosutinib (Src/Abl inhibitor) in this panel, as it was previously identified by two independent studies as an inhibitor of ZIKV replication (30, 31). All inhibitors were used at concentrations that retained at least 90% cell viability (CC_10_) relative to DMSO (vehicle control) after 72 h of treatment, as determined by an ATP-based luminescent cell viability assay (Table 1). A549 cells are commonly used to study ZIKV infections as they show similar ZIKV replication and responses to mosquito C6/36 and the human placental trophoblast cell line JEG-3 (32, 33). We infected A549 cells with ZIKV under multi-cycle conditions [multiplicity of infection (MOI) = 0.01 plaque-forming units (PFU)/cell] to probe for SMKI effects on every step of the viral life cycle. The compounds were added immediately after virus adsorption, and viral titers were measured at the peak of replication (i.e., 72 hpi). Only treatment with either Dabrafenib (B-Raf/c-Raf inhibitor) or Regorafenib (B-Raf/c-Raf, RET, VEGFR, and c-Kit inhibitor) significantly inhibited ZIKV replication (~100-fold and 10-fold reduction, respectively) (Fig. 1A). Surprisingly, Bosutinib treatment did not reduce viral titers under these conditions. In addition, we examined ZIKV spread by immunofluorescence staining for ZIKV E protein. In contrast to the efficient viral spread in DMSO-treated cells, very few cells were positive for E protein in Dabrafenib-treated cells (Fig. 1B); Regorafenib also significantly reduced viral spread but was less efficient than Dabrafenib. Consistent with our viral titer data, Bosutinib treatment had a minimal impact on ZIKV spread. We then compared viral titers at 24, 48, and 72 hpi to better understand the effect of these SMKIs on viral replication kinetics. Both Dabrafenib and Regorafenib caused a moderate decrease in viral titers as early as 24 hpi; however, stronger titer reductions were observed at 48 hpi and were even more robust at 72 hpi, the peak of replication (Fig. 1C). No significant reduction in viral titers was observed for Bosutinib at any time point.

**TABLE 1:** SMKI targets and CC_10_ in A549 and vero cells^*a*^

Inhibitor	Target(s)	CC_10_(A549) [µM]	CC_10_(Vero) [µM]
Acalabrutinib	BTK	0.5	ND
Afatinib	EGFR, HER2	5	ND
Avapritinib	PDGFRα,	0.125	ND
Axitinib	VEGFR1/2/3, PDGFRβ, c-Kit	10	ND
Belvarafenib	c-Raf, B-Raf, B-Raf^V600E^	0.25	ND
Bosutinib	Src, Abl	5	ND
Cabozantinib	VEGFR2, c-Met, RET, c-Kit	1	ND
Dabrafenib	B-Raf, B-Raf^V600E^, c-Raf	10	10
Defactinib	FAK	5	ND
Encorafenib	B-Raf^V600E^	0.5	ND
Ibrutinib	BTK	0.5	ND
Larotrectinib	TrkA/B/C	0.125	ND
Neratinib	HER2, EGFR	0.01	ND
Nilotinib	Bcr-Abl	1	ND
Regorafenib	RET, c-Raf, VEGFR1/2/3, B-Raf, PDGFRβ, c-Kit	2.5	1
Saracatinib	SFKs (Src, Lck, Yes, Fyn, Fgr, Blk), EGFR	0.125	ND
Sorafenib	c-Raf, B-Raf, VEGFR2	2.5	ND
Y15	FAK	5	ND

^*a*^ CC10, 10% cytotoxic concentration (90% viability); ND, not determined in this study. BTK, Bruton’s tyrosine kinase; B-Raf^V600E^, oncogenic B-Raf mutant; EGFR, epidermal growth factor receptor; FAK, focal adhesion kinase; HER2, human epidermal growth factor receptor 2; PDGFR, platelet-derived growth factor receptor; SFKs, Src family kinases; VEGFR, vascular endothelial growth factor receptor.

We next assessed the impact of the infectious dose of ZIKV on the observed antiviral effects of the tested inhibitors. Cells were infected with ZIKV at a 100-fold higher MOI (MOI = 1 PFU/cell) than in our previous experiments. Both Dabrafenib and Regorafenib remained highly effective at inhibiting virus production at this MOI (Fig. 1D). Surprisingly, Bosutinib also impaired ZIKV replication under these conditions, though not as strongly as Dabrafenib and Regorafenib. These results indicate that, unlike bosutinib, the antiviral effects of Dabrafenib and Regorafenib are MOI-independent.

Because Dabrafenib and Regorafenib target multiple kinases, we used target deconvolution by screening additional SMKIs with partially overlapping specificities to identify the kinase(s) mediating the observed antiviral activities. We compared the effects of Belvarafenib (B-Raf/c-Raf inhibitor), Encorafenib (B-Raf^V600E^ inhibitor), Axitinib (PDGFR, VEGFR, and c-Kit inhibitor), Cabozantinib (c-Kit, c-Met, and RET inhibitor), and Sorafenib (predecessor compound of Regorafenib) on ZIKV-infected cells to treatment with Dabrafenib and Regorafenib. Neither Belvarafenib nor Encorafenib affected ZIKV replication (Fig. 1E). Interestingly, Axitinib, which inhibits several kinases targeted by Regorafenib but not B-Raf or c-Raf, did not significantly reduce viral titers. Similarly, Cabozantinib treatment did not affect ZIKV titers, excluding a role for RET in ZIKV replication. Sorafenib, which is less potent than Regorafenib, also exhibited a reduced but significant inhibition of ZIKV replication. These data suggest that the observed antiviral effects of Dabrafenib and Regorafenib were likely due to reduced B-Raf and/or c-Raf kinase activity.

To confirm our findings in another mammalian cell line of non-cancerous origin, we used the well-established Vero cells, which are also highly permissive for ZIKV infection. ZIKV-infected cells treated with either Dabrafenib or Regorafenib also exhibited a significant reduction in ZIKV titers (Fig. 1F). Titer reductions in Vero cells were less robust as those observed in A549 cells, possibly due to the type I interferon (IFN) deficiency of Vero cells (34). Surprisingly, neither Dabrafenib nor Regorafenib treatment had a negative impact on replication of the closely related DENV2 (Fig. 1G), suggesting differential roles for Raf kinases in ZIKV and DENV infections.

### Dabrafenib and Regorafenib target ZIKV infection at a post-entry stage

**Fig 2:**
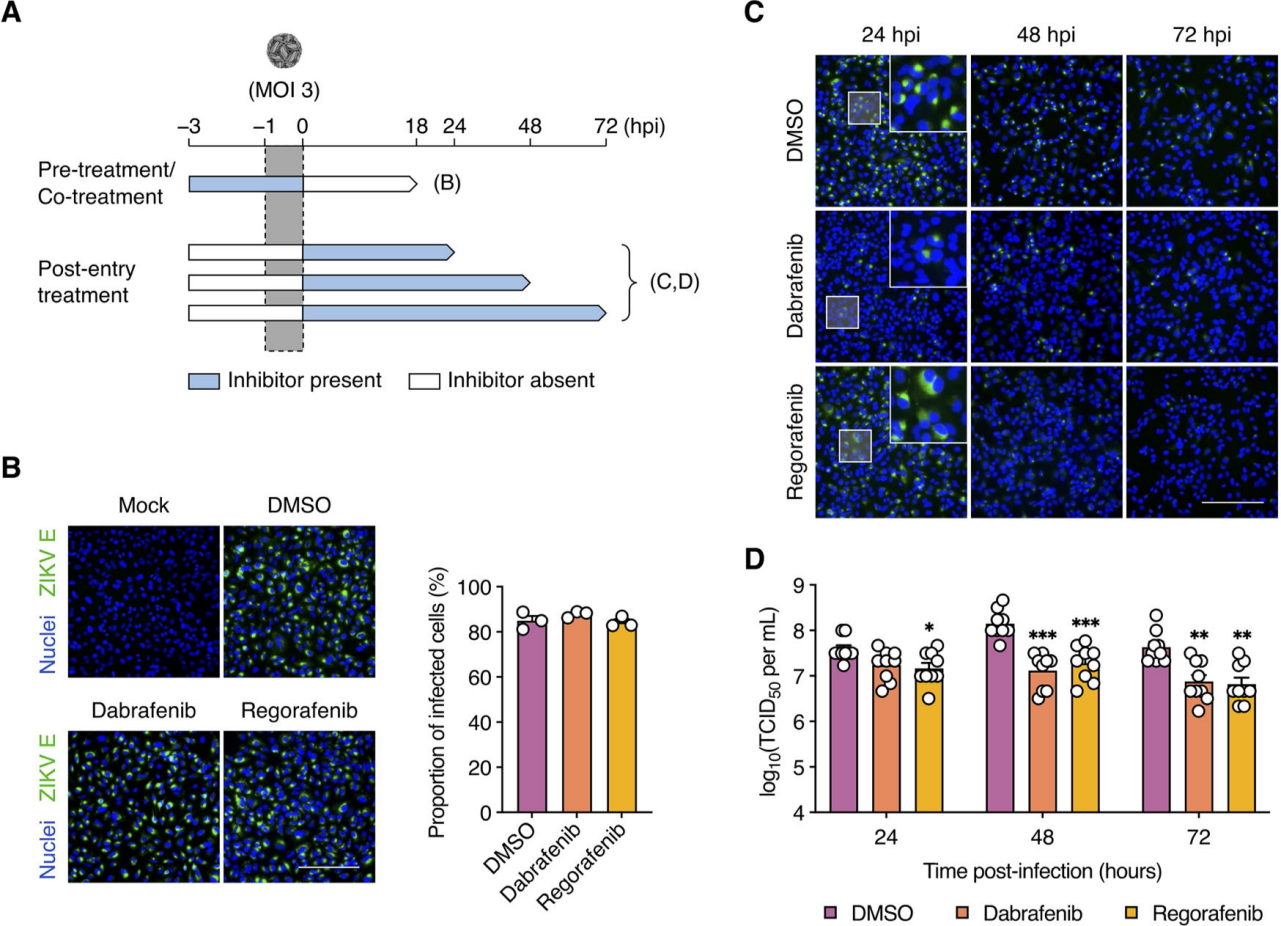
Dabrafenib and Regorafenib target ZIKV infection at the post-entry stage. (**A**) Schematic representation of the time-of-addition assays. (**B**) A549 cells were pre-treated with DMSO (vehicle control), Dabrafenib (10 µM), or Regorafenib (2.5 µM) for 2 h prior to infection with ZIKV (MOI = 3 PFU/cell) as well as during the adsorption period. ZIKV infection was visualized at 18 hpi by immunofluorescence staining for ZIKV E protein (green). Nuclei were stained with Hoechst 33342 (blue). Scale bar, 200 µm. Images are representative of three independent experiments. Bar graph shows the proportions of infected cells determined from immunofluorescence images (one per experiment) using ImageJ. (**C and D**) A549 cells were infected with ZIKV (MOI = 3 PFU/cell) and subsequently treated with DMSO, Dabrafenib (10 µM), or Regorafenib (2.5 µM). (**C**) ZIKV infection was visualized at 24, 48, and 72 hpi by immunofluorescence staining for ZIKV E protein (green). Nuclei were stained with Hoechst 33342 (blue). Scale bar, 200 µm. Images are representative of three independent experiments. (**D**) Viral titers in the supernatant were determined at 24, 48, and 72 hpi by endpoint dilution assay. Data are expressed as mean ± SEM from three independent experiments with three biological replicates per experiment. *, *P* < 0.05; **, *P* < 0.01; ***, *P* < 0.001 (Mann–Whitney *U* test vs “DMSO”).

To define the steps of the ZIKV infection cycle affected by Dabrafenib and Regorafenib, we performed time-of-addition assays under single-cycle conditions (MOI = 3 PFU/cell) (Fig. 2A). To study effects on viral entry, A549 cells were treated with inhibitors for 2 h prior to virus addition (pre-treatment) as well as during the viral adsorption period (co-treatment). At 18 hpi, ZIKV infection was detected by immunofluorescence staining for E protein. We did not observe any reduction in the proportion of infected cells following combined pre-treatment and co-treatment with either Dabrafenib or Regorafenib compared to DMSO-treated cells (Fig. 2B), suggesting that neither inhibitor acts on ZIKV entry.

Next, we assessed the effects on post-entry events by adding Dabrafenib or Regorafenib after virus adsorption. At 24, 48, and 72 hpi, we assessed E protein staining and virus production. Although no significant difference in E protein staining was observed following Regorafenib treatment at 24 hpi, its pattern was more diffuse than in DMSO-treated cells (Fig. 2C). Intriguingly, we observed a sharp reduction in E protein by 48 hpi until it was largely undetectable by 72 hpi in Regorafenib-treated cells. In contrast, only a few cells were positive for E protein staining at these time points following Dabrafenib treatment. Surprisingly, a moderate yet significant reduction in viral titers was observed at 24 hpi in Regorafenib-treated cells, but not in Dabrafenib-treated cells (Fig. 2D). However, both inhibitors caused strong reductions in viral titer at 48 and 72 hpi suggesting that Dabrafenib and Regorafenib inhibit ZIKV replication following viral entry into host cells.

### Dabrafenib, but not Regorafenib, impairs ZIKV genome replication

**Fig 3:**
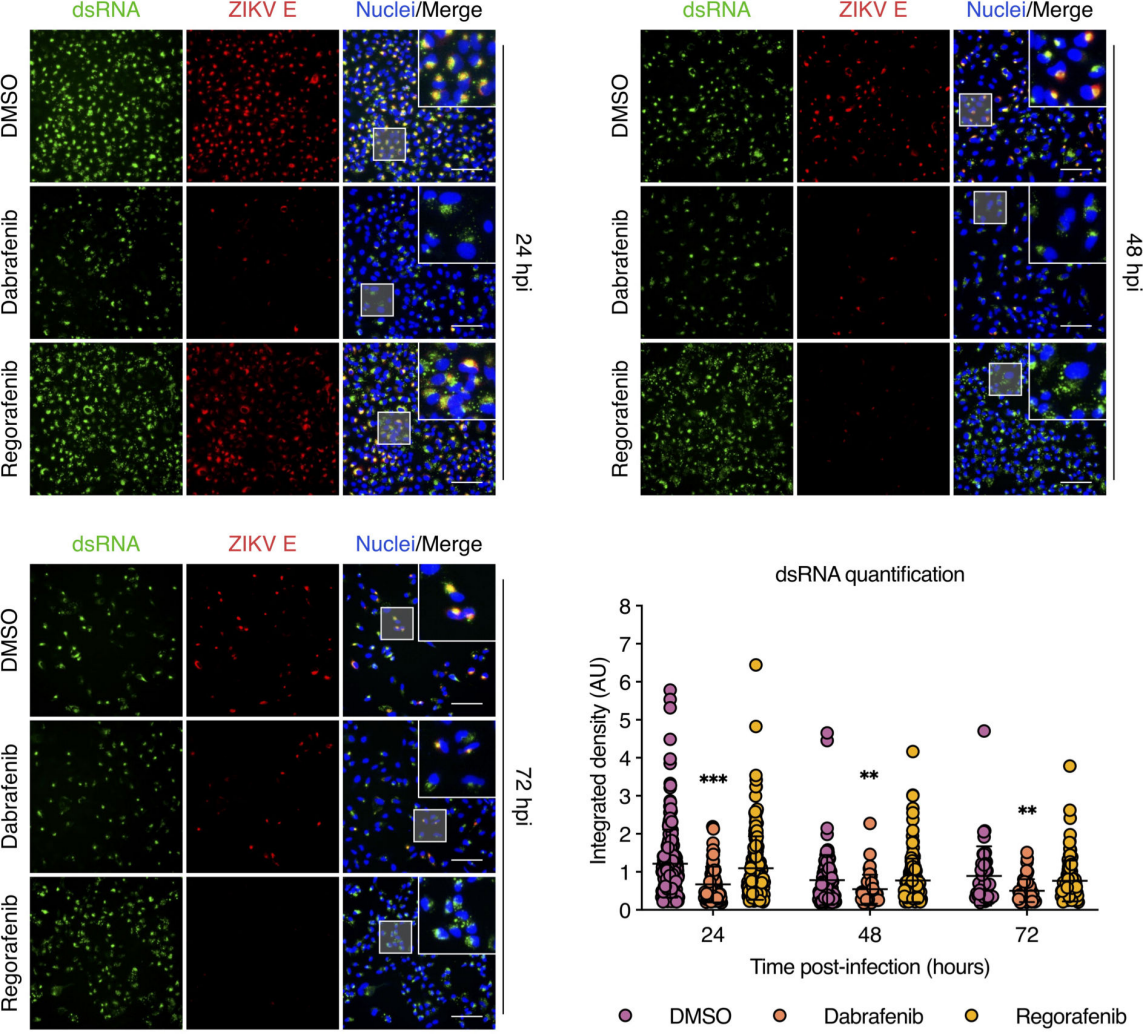
Dabrafenib, but not Regorafenib, reduces viral dsRNA levels. A549 cells were infected with ZIKV (MOI = 3 PFU/cell) and subsequently treated with DMSO (vehicle control), Dabrafenib (10 µM), or Regorafenib (2.5 µM). ZIKV infection was visualized at 24, 48, and 72 hpi by immunofluorescence staining for ZIKV E protein (red) and dsRNA (green). Nuclei were stained with Hoechst 33342 (blue). Scale bars, 100 µm. Dot plot shows image-based quantification of the integrated density of dsRNA in individual cells. Data are expressed as mean ± SEM. Data from one of three independent experiments with similar results are shown. *, *P* < 0.05; **, *P* < 0.01; ***, *P* < 0.001 (unpaired Student’s *t* test vs “DMSO”).

To better understand the antiviral effects of Dabrafenib and Regorafenib, we assessed their impact on ZIKV genome replication. At 24, 48, and 72 hpi, ZIKV genome replication was detected by dsRNA-specific immunofluorescence staining in the presence or absence of either Dabrafenib or Regorafenib. E protein staining was used as a marker for viral protein synthesis. Compared to DMSO-treated cells, we observed a significant reduction in dsRNA staining in Dabrafenib-treated cells at 24, 48, and 72 hpi that correlated with E protein staining (Fig. 3). In contrast, no decrease in dsRNA staining was observed in Regorafenib-treated cells at any time point; however, dsRNA staining was more diffuse throughout the cytoplasm, most notably at 48 hpi. Interestingly, we observed a significant reduction in E protein staining by 48 hpi despite high levels of dsRNA detected in Regorafenib-treated cells, suggesting effects on translation and/or protein stability but not genome replication. These data indicate that Dabrafenib, but not Regorafenib, interferes with the ZIKV RNA replication process.

**Fig 4:**
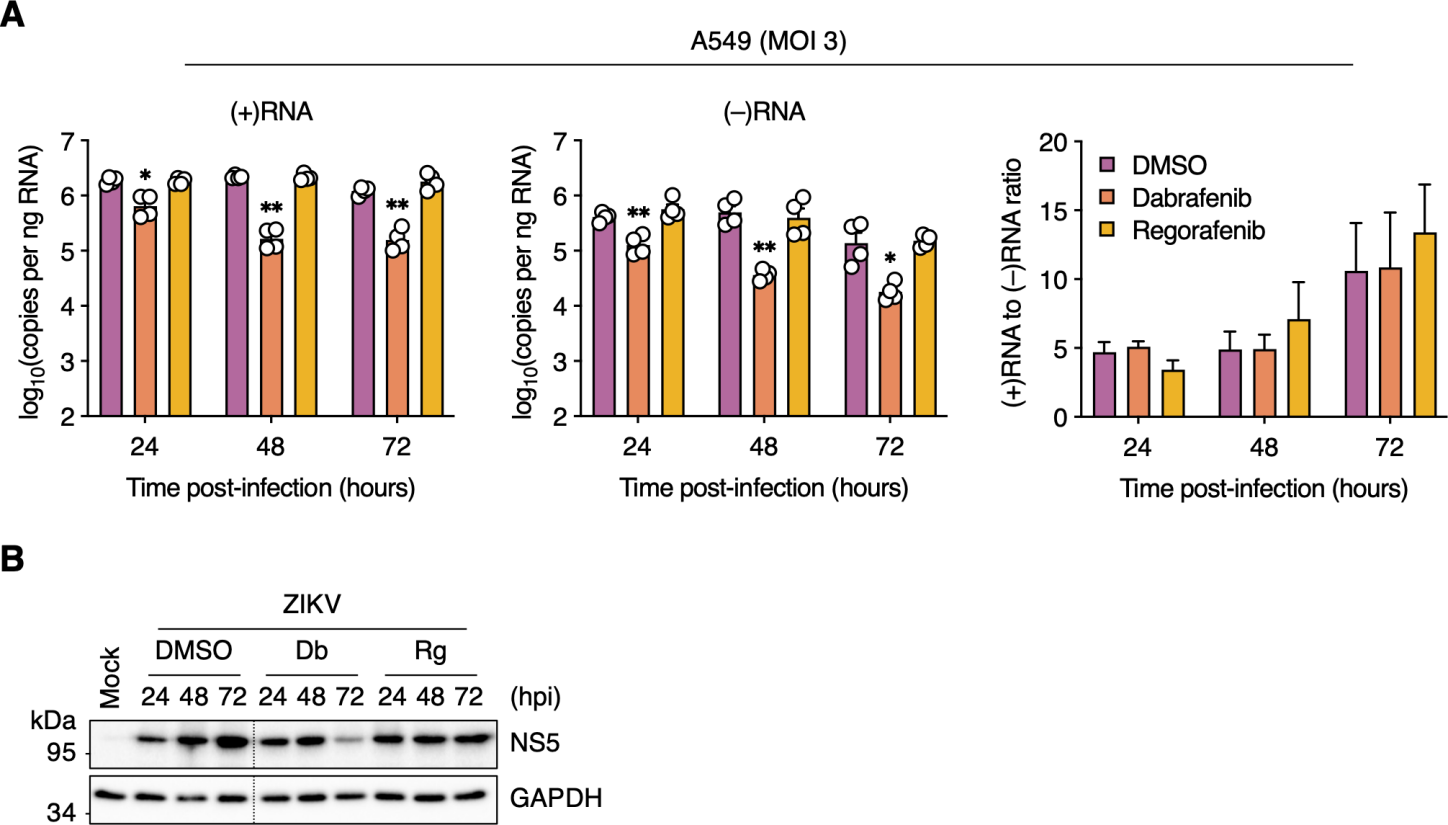
Dabrafenib, but not Regorafenib, attenuates viral RNA synthesis. (**A**) A549 cells were infected with ZIKV (MOI = 3 PFU/cell) and subsequently treated with DMSO (vehicle control), Dabrafenib (10 µM), or Regorafenib (2.5 µM). Total cell-associated RNA was isolated at 24, 48, and 72 hpi, and analyzed by positive strand-specific RT-qPCR (upper left panel) and negative strand-specific RT-qPCR (upper right panel). Lower left panel: Results shown in the upper panels plotted as the ratio of positive-strand RNA [(+)RNA] to negative-strand RNA [(−)RNA]. Data are expressed as mean ± SEM from two independent experiments with two biological replicates per experiment. *, *P* < 0.05; **, *P* < 0.01 (two-way ANOVA vs “DMSO”). (**B**) A549 cells were mock-infected or infected with ZIKV (MOI = 3 PFU/cell) and were left untreated (Untr) or were treated with DMSO (vehicle control), Dabrafenib (Db; 10 µM), or Regorafenib (Rg; 2.5 µM) for the indicated durations. Whole-cell lysates were analyzed by Western blotting with antibodies against ZIKV NS5 and GAPDH (loading control). Black dotted lines indicate removal of portions of the blots for clarity.

We used a strand-specific RT-qPCR assay to further dissect the observed reduction in genome replication in Dabrafenib-treated cells by assessing the effects on negative-strand RNA [(−)RNA] and positive-strand RNA [(+)RNA] synthesis. We found that Dabrafenib significantly reduced the levels of both (−)RNA and (+)RNA at all time points as early as 24 hpi and more robustly at 48 and 72 hpi (Fig. 4A). As expected, Regorafenib treatment did not affect synthesis of either strand. Furthermore, the ratio of (+)RNA to (−)RNA did not change in Dabrafenib-treated cells (Fig. 4A), indicating a uniform impact on the synthesis of both viral RNA species and suggesting an effect on the viral polymerase NS5. Although there was a steady increase in NS5 levels from 24 to 72 hpi in DMSO-treated cells, we observed a reduction in NS5 levels from 24 to 72 hpi in Dabrafenib-treated cells. In contrast, we did not detect a change in NS5 levels from 24 to 72 hpi in Regorafenib-treated cells (Fig. 4B). Taken together, these data indicate that Dabrafenib and Regorafenib differentially regulate B-Raf and c-Raf signaling to affect ZIKV genome replication.

### Regorafenib reduces ZIKV E protein, but not NS1, levels

Given that Regorafenib treatment reduced ZIKV E protein staining but not viral RNA replication, we assessed its effect on viral protein synthesis. Sorafenib, a structurally similar SMKI to Regorafenib, causes PERK/eIF2α-mediated translational inhibition in various tumor cell lines (35, 36). One of the hallmarks of sustained eIF2α-mediated stress responses is formation of stress granules (SGs), cytoplasmic aggregates of stalled 48S translation preinitiation complexes. Because Axitinib does not inhibit B-Raf or c-Raf but does inhibit the other kinases targeted by Regorafenib, it was included as a control.

**Fig 5:**
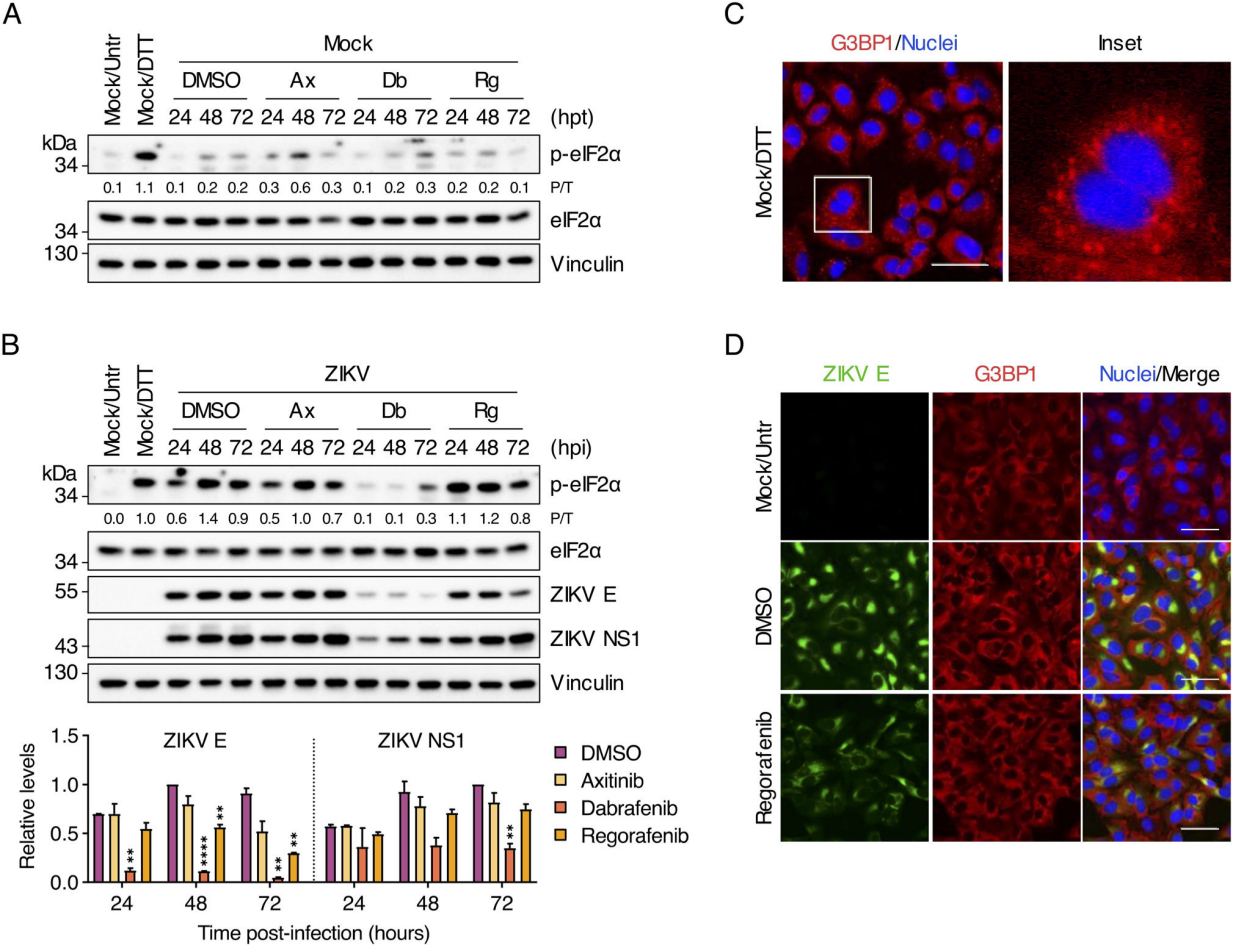
Regorafenib reduces ZIKV E protein, but not NS1, levels and does not activate the integrated stress response. (**A**) A549 cells were either left untreated (Untr) or were treated with DMSO (vehicle control), Axitinib (Ax; 10 µM), Dabrafenib (Db; 10 µM), or Regorafenib (Rg; 2.5 µM) for the indicated durations. As a positive control, cells were treated with DTT (2 mM) for 30 min prior to harvesting. Whole-cell lysates were analyzed by Western blotting with antibodies against phospho-eIF2α (Ser51), total eIF2α, and vinculin (loading control). (**B**) A549 cells were mock-infected or infected with ZIKV (MOI = 3 PFU/cell) and were left untreated (Untr) or were treated with DMSO (vehicle control), Axitinib (Ax; 10 µM), Dabrafenib (Db; 10 µM), or Regorafenib (Rg; 2.5 µM) for the indicated durations. As a positive control, cells were treated with DTT (2 mM) for 30 min prior to harvesting. Whole-cell lysates were analyzed by Western blotting with antibodies against phospho-eIF2α (Ser51), total eIF2α, ZIKV E protein, and vinculin (loading control). Ratios of phospho-eIF2α to total eIF2α (P/T) and the relative levels of ZIKV E protein and ZIKV NS1 were determined by densitometric analysis. The latter are shown as bar graphs and are expressed as mean ± standard deviation (SD). **, *P* < 0.01; ****, *P* < 0.0001 (multiple *t* test with Holm–Šidák correction vs “DMSO”). Blots in (**A**) and (**B**) are representative of two independent experiments. (**C**) A549 cells were treated with DTT (2 mM) for 30 min prior to fixation and SGs were visualized by immunofluorescence staining for G3BP1 (red). Nuclei were stained with Hoechst 33342 (blue). Scale bar, 50 µm. (**D**) A549 cells were mock-infected or infected with ZIKV (MOI = 3 PFU/cell) and were left untreated (Untr) or treated with DMSO (vehicle control) or Regorafenib (2.5 µM) for 24 h. ZIKV infection and SGs were visualized by immunofluorescence staining for ZIKV E protein (green) and G3BP1 (red), respectively. Scale bars, 50 µm. Images are representative of two independent experiments.

A minimal increase in eIF2α phosphorylation was detected in mock-infected, Axitinib-treated and Dabrafenib-treated cells at 48 and 72 h post-treatment, respectively (Fig. 5A). No eIF2α phosphorylation was observed in either DMSO-treated or Regorafenib-treated cells. As expected, a robust level of eIF2α phosphorylation was present in cells treated with the reducing agent dithiothreitol (DTT), an inducer of ER stress and PERK activator (37).

ZIKV infection induced eIF2α phosphorylation by 24 hpi, peaking at 48 hpi, followed by a slight decrease at 72 hpi in DMSO-treated cells (Fig. 5B). Although Axitinib-treated cells exhibited similar levels of ZIKV-induced eIF2α phosphorylation to DMSO-treated cells, Dabrafenib-treated cells exhibited reduced levels of eIF2α phosphorylation throughout the course of infection (Fig. 5B). In contrast, Regorafenib treatment induced high levels of eIF2α phosphorylation by 24 hpi that persisted until 48 hpi, after which they slightly declined. Under these conditions, ZIKV E protein levels were comparable in DMSO-treated and Axitinib-treated cells (Fig. 5B). Treatment with either Regorafenib or, to a lesser extent, Dabrafenib resulted in a decrease in ZIKV E protein expression. Surprisingly, no decrease in NS1 levels from 24 to 72 hpi was detected under any condition.

### Regorafenib does not induce SG formation during ZIKV infection

Like other flaviviruses, ZIKV progressively inhibits eIF2α-dependent SG formation with time after infection (38–41). SGs were readily detected by immunofluorescence staining for the SG marker, G3BP1, in mock-infected cells treated with DTT (Fig. 5C). However, despite observing high levels of phospho-eIF2α in Regorafenib-treated cells at early times after infection (i.e., 24 hpi), we did not observe the formation of SGs (Fig. 5D). As expected, we also did not detect any SGs in DMSO-treated ZIKV-infected cells. These data suggest that the observed reduction in ZIKV E by Regorafenib treatment is not due to eIF2α-induced SG formation and subsequent suppression of viral protein synthesis.

### Regorafenib treatment results in inefficient ZIKV NS1 secretion

**Fig 6:**
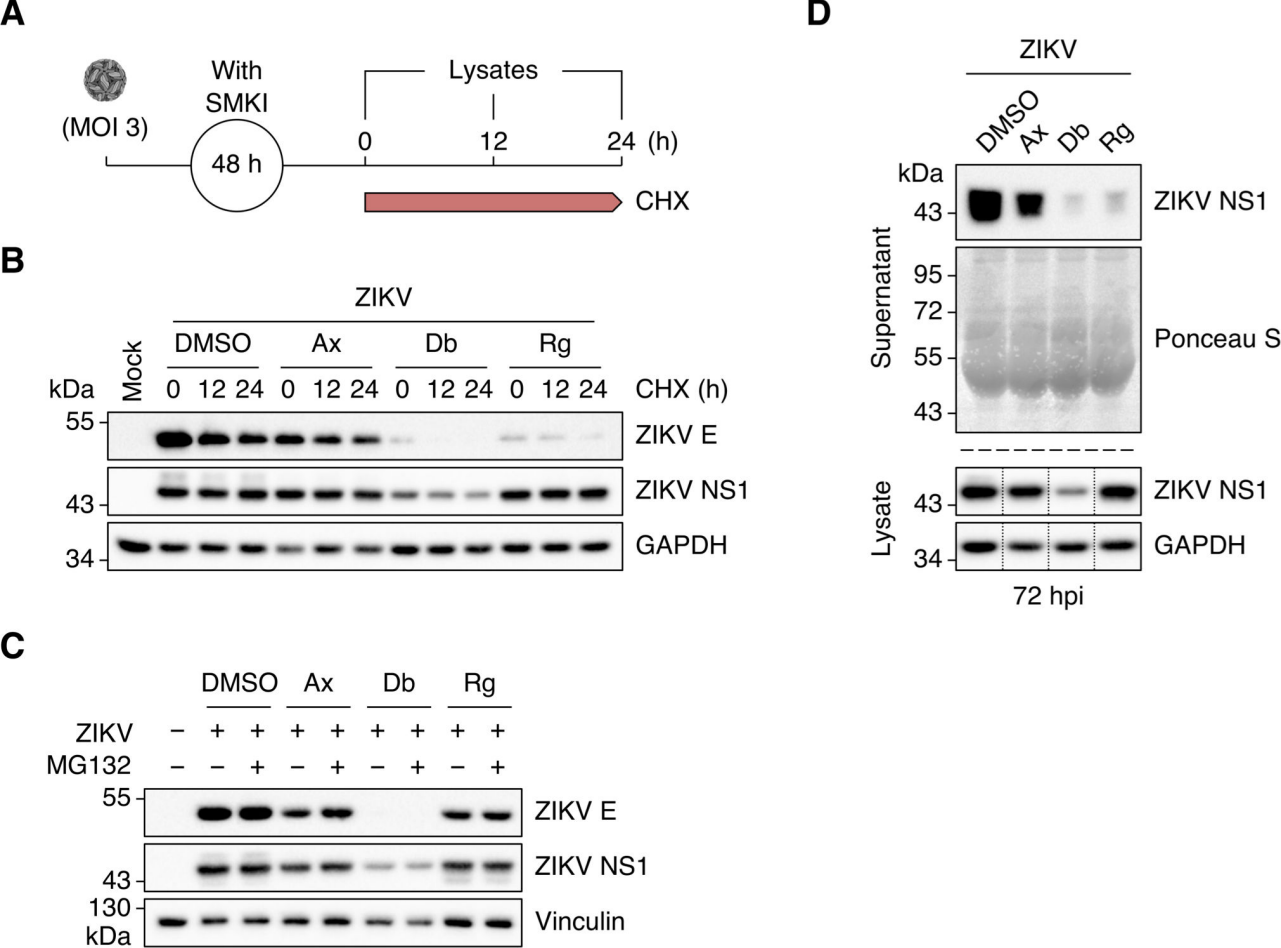
Regorafenib treatment results in inefficient ZIKV NS1 secretion. (**A**) Schematic representation of the cycloheximide (CHX)–chase experiment. (**B**) A549 cells were mock-infected or infected with ZIKV (MOI = 3 PFU/cell) and subsequently treated with DMSO (vehicle control), Axitinib (Ax; 10 µM), Dabrafenib (Db; 10 µM), or Regorafenib (Rg; 2.5 µM). At 48 hpi, cells were exposed to cycloheximide (10 µg/mL) and chased for 0, 12, and 24 h. Whole-cell lysates were analyzed by Western blotting with antibodies against ZIKV E protein, ZIKV NS1, and GAPDH (loading control). Blots are representative of two independent experiments. (**C**) A549 cells were mock-infected or infected with ZIKV (MOI = 3 PFU/cell) and subsequently treated with DMSO (vehicle control), Axitinib (Ax; 10 µM), Dabrafenib (Db; 10 µM), or Regorafenib (Rg; 2.5 µM). At 56 hpi, cells were exposed to MG132 (10 µM) or left untreated. Whole-cell lysates were collected at 72 hpi and analyzed by Western blotting with antibodies against ZIKV E protein, ZIKV NS1, and vinculin (loading control). (**D**) A549 cells were infected with ZIKV (MOI = 3) and subsequently treated with DMSO (vehicle control), Axitinib (Ax; 10 µM), Dabrafenib (Db; 2.5 µM), or Regorafenib (Rg; 2.5 µM). Culture supernatants were collected at 72 hpi, precipitated with TCA and analyzed by Western blotting with an antibody against ZIKV NS1. Ponceau S staining served as a loading control. Whole-cell lysates were processed in parallel and analyzed by Western blotting with antibodies against ZIKV NS1 and GAPDH (loading control). Black dotted lines indicate removal of portions of the blots for clarity.

We were intrigued by the observation that NS1 levels remained elevated in cells treated with Regorafenib, even though E protein expression was impaired. To determine if the Regorafenib-mediated decline in E protein we observed was due to increased protein turnover, we exposed SMKI-treated ZIKV-infected cells to the translation inhibitor cycloheximide (CHX) at 48 hpi and chased them for 0, 12, or 24 h (Fig. 6A). We observed a time-dependent reduction in E protein abundance under all conditions (Fig. 6B). In contrast, the levels of NS1 were stable under all conditions. To rule out Regorafenib-induced proteasomal degradation of viral proteins, we exposed SMKI-treated ZIKV-infected cells to the proteasomal inhibitor MG132 (10 µM) or DMSO at 56 hpi and whole-cell lysates were collected at 72 hpi. We did not observe a significant increase in either E or NS1 protein levels following proteasomal inhibition (Fig. 6C). Taken together, the data suggest that Regorafenib does not inhibit proteasome-mediated viral protein turnover.

NS1 dimers are targeted from the ER lumen to viral replication sites and the plasma membrane, whereas NS1 tetramers and hexamers are extracellularly secreted (42, 43). To determine whether the accumulation of NS1 in Regorafenib-treated cells was due to inefficient secretion of the protein, we compared intracellular and supernatant NS1 levels. As expected, comparable levels of intracellular NS1 were present in cells treated with DMSO, Axitinib, and Regorafenib (Fig. 6D). Intriguingly, very little NS1 was released from cells treated with Regorafenib. NS1 secretion was also somewhat reduced in Axitinib-treated cells. As expected, low levels of both intracellular and secreted NS1 were observed after Dabrafenib treatment. Our data suggest that Regorafenib treatment, in particular, results in inefficient NS1 secretion due to intracellular retention.

### Regorafenib alters ER morphology in ZIKV-infected cells and limits viral egress

**Fig 7:**
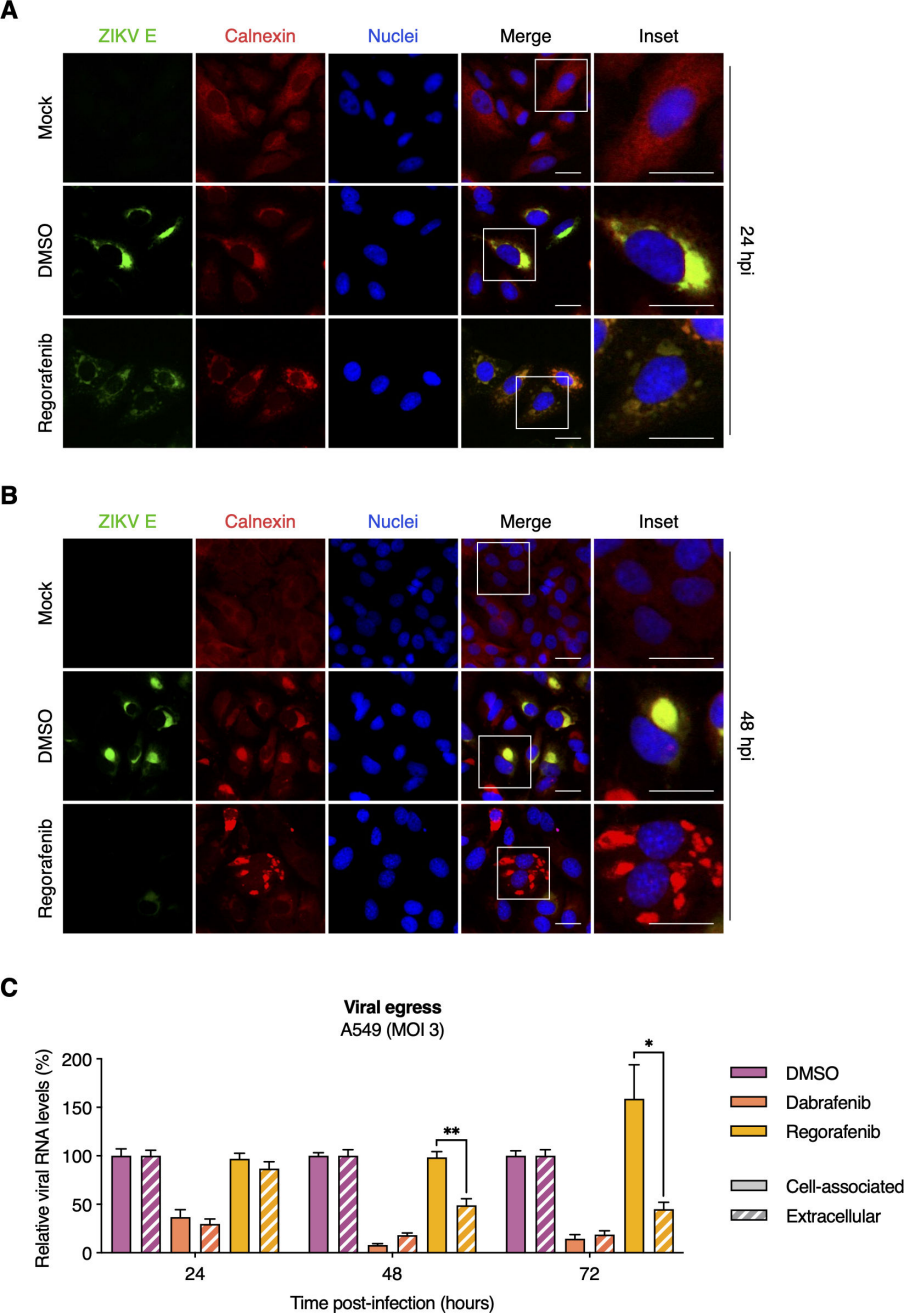
Regorafenib alters ER morphology in ZIKV-infected cells and limits viral egress. (**A and B**) A549 cells were mock-infected or infected with ZIKV (MOI = 3 PFU/cell) and subsequently treated with DMSO (vehicle control) or Regorafenib (2.5 µM). At 24 hpi (**A**) and 48 hpi (**B**), ZIKV infection and ER were visualized by immunofluorescence staining for ZIKV E protein (green) and calnexin (red), respectively. Nuclei were stained with Hoechst 33342 (blue). Scale bars, 25 µm. Images are representative of two independent experiments. (**C**) A549 cells were infected with ZIKV (MOI = 3 PFU/cell) and subsequently treated with DMSO (vehicle control), Dabrafenib (10 µM), or Regorafenib (2.5 µM). At 24, 48, and 72 hpi, total cell-associated RNA and extracellular viral RNA were isolated, and quantified by RT-qPCR. Data were normalized to DMSO control, and are expressed as mean ± SEM from two independent experiments with two biological replicates per experiment. *, *P* < 0.05; **, *P* < 0.01 (unpaired Student’s *t* test, “cell associated” vs “extracellular”).

We next determined whether the observed E protein depletion and NS1 accumulation in Regorafenib-treated cells was associated with disruption of the ER, the site of viral replication and virion assembly. In mock-infected cells, we observed typical and even distribution of the ER marker, calnexin. As expected, we readily observed significant alterations of the ER in ZIKV-infected cells where ZIKV E and calnexin also co-localized in the perinuclear regions by 24 hpi; this was the case in both DMSO-treated and Regorafenib-treated cells (Fig. 7A). However, calnexin staining indicated progressively fragmented ER morphology in Regorafenib-treated cells but not DMSO-treated cells (Fig. 7A and B). Consistent with our earlier results, ZIKV E was minimally detectable at 48 hpi in Regorafenib-treated cells.

To assess the effect of Regorafenib on ZIKV egress efficiency, we compared the extracellular levels of viral RNA relative to cell-associated viral RNA as a surrogate for viral release. Total cell-associated RNA and viral RNA in the supernatant of ZIKV-infected (MOI = 3 PFU/cell) DMSO- or SMKI-treated cells were isolated at 24, 48, and 72 hpi. Viral (+)RNA was quantified by RT-qPCR and the relative levels in each fraction were compared. We did not detect a difference between the fractions from DMSO-treated or Dabrafenib-treated cells (Fig. 7C). However, we observed a significant reduction (>50%) in extracellular viral RNA compared to cell-associated viral RNA after Regorafenib treatment. Moreover, the difference was even greater by 72 hpi. Although the extracellular fraction was comparable to that observed at 48 hpi, the cell-associated fraction was significantly greater at 72 hpi (>50%). Taken together, these results suggest that Regorafenib treatment alters ER morphology and limits ZIKV assembly and/or egress.

## DISCUSSION

Currently, there are no licensed ZIKV vaccines or antivirals (44); therefore, we sought to identify promising host-directed antivirals for use during outbreaks to mitigate the spread of ZIKV infections and the associated disease burden. We screened the antiviral activities of a panel of FDA-approved SMKIs that we recently identified as potent inhibitors of seasonal and pandemic influenza A viruses (27, 28). Of the 13 inhibitors tested, we identified Dabrafenib and Regorafenib (Raf kinase inhibitors) as potent antagonists of ZIKV replication that reduced viral titers by ~100-fold and ~10-fold, respectively. Although both inhibitors target B-Raf and c-Raf, they seem to act at different steps of the ZIKV infection cycle. This is consistent with these kinases mediating different cellular processes in a stimuli-specific manner; as is the case with many non-receptor tyrosine kinases. We found that Dabrafenib inhibits viral genome replication and thereby affects subsequent protein expression, ultimately resulting in reduced viral progeny. In contrast, Regorafenib inhibits ZIKV protein expression and viral egress. Our target deconvolution studies point to a combined role for both B-Raf and c-Raf in mediating the antiviral effects of Dabrafenib and Regorafenib. This is further supported by the fact that SMKIs that do not target Raf kinases (Axitinib and Cabozantinib) had no appreciable effect on ZIKV infection. Surprisingly, neither Belvarafenib (B-Raf/c-Raf inhibitor) nor Encorafenib (B-Raf^V600E^ inhibitor) affected ZIKV infection. Despite having an IC_50_ similar to Regorafenib against B-Raf and c-Raf, these inhibitors exhibited lower CC_10_ (i.e., higher toxicity) and were therefore used at lower concentrations which may account for the lack of efficacy we observed in our study. Consistent with recent reports of antiviral activity against several viruses including ZIKV (45–48), we observed a significant reduction in ZIKV titers following treatment with Sorafenib, a predecessor compound of Regorafenib. ZIKV replicates to higher titers in the type I IFN-deficient Vero cells than in A549 cells. We observed similar reductions in ZIKV-infected Vero cells treated with either Dabrafenib or Regorafenib, confirming that the effect of these inhibitors is type I IFN-independent and cell type-independent. Unlike most proteins, kinase conservation and homology are typically restricted to their functional domains including kinase domains, ATP-binding sites and protein-protein interaction domains and are evolutionarily conserved across different species including flies and worms (49). In related species or genera, functional conservation is extremely high among kinase orthologs. Both C-Raf/Raf-1 and B-Raf are highly conserved between humans and African green monkeys (source of Vero cells) at 100% and 94% amino acid identity, respectively. Therefore, it is expected that these inhibitors would be effective against kinase orthologs from other species with similar antiviral potency, providing that other pathway components are also conserved.

Surprisingly, DENV infection was not affected by treatment with either Dabrafenib or Regorafenib. The role of Raf–MEK–ERK signaling in DENV infection is not fully understood and may be species-specific based on studies using MEK inhibitors (50, 51). Contrary to our observations, pretreatment of DENV2-infected cells with a preclinical c-Raf inhibitor (K039) was reported to modestly (~7-fold) reduce viral titers (52). However, the selectivity of K039 has not been validated and it is not clear if other kinases are targeted by this compound. Additionally, that study used the DENV2 laboratory strain New Guinea C (NGC), whereas our studies were conducted using a contemporary isolate. It is therefore possible that strain-specific as well as serotype-specific differences may influence the role Raf kinases play in DENV infections.

A recent study reported that whereas inhibition of EGFR-mediated Ras–Raf–MEK–ERK signaling limited ZIKV entry at early time points, inhibition of this pathway at later time points had little to no impact on ZIKV replication (48). Contrary to these results, we found that pre-treatment (including during virus inoculation) with either Dabrafenib or Regorafenib had no appreciable effect on viral infectivity, indicating neither of these inhibitors affects ZIKV entry. However, a clear reduction in viral titers was observed as early as 24 hpi when cells were treated following inoculation, suggesting effects on later steps of ZIKV infection.

A striking difference between Dabrafenib and Regorafenib was their impact on ZIKV genome replication. ZIKV-infected cells treated with Dabrafenib, but not Regorafenib, exhibited a markedly reduced level of detectable dsRNA replication intermediates. Moreover, we found a reduction in the synthesis of both (−)RNA and (+)RNA. Flavivirus genome replication is asymmetric with many more copies of positive-strand RNA synthesized from the negative-strand RNA template (53). However, despite the reduction in detectable (+)RNA and (−)RNA, their ratio was not affected by Dabrafenib treatment, suggesting a direct effect on the NS5 viral polymerase. The reduced levels of NS5 detected in Dabrafenib-treated cells compared to DMSO-treated cells may partially explain this effect. Alternatively, NS5 polymerase activity may be affected either directly via reduced phosphorylation or indirectly by interference with replication complex formation by Dabrafenib treatment. Dabrafenib-mediated reduction in (+)RNA synthesis likely drives the overall reduction in detectable ZIKV E and NS1 proteins we observed.

Although Regorafenib does not directly affect genome replication in ZIKV-infected cells, ZIKV E protein was almost undetectable by 48 hpi in Regorafenib-treated cells. Translation inhibition via stress-induced phosphorylation of eIF2α is a well-established antiviral mechanism to which many flaviviruses have evolved countermeasures (54–57). A hallmark of eIF2α-mediated translational repression is the formation of SGs, wherein stalled 48S translation preinitiation complexes form distinct cytoplasmic aggregates and result in global translational shutoff. Many flaviviruses either actively suppress or prevent eIF2α phosphorylation. One mechanism is to avoid viral dsRNA detection by the eIF2α kinase, PKR, by sequestrating replication complexes in ER membrane vesicles (57, 58). Excessive early replication may lead to inefficient formation of these vesicles and allow cytosolic exposure of viral dsRNA, resulting in eIF2α phosphorylation and subsequent SG formation (59). Although there are conflicting reports regarding the induction of eIF2α phosphorylation during ZIKV infection, ZIKV actively suppresses SG formation partly by decoupling eIF2α-dependent SG formation (38–41). Accordingly, Regorafenib treatment did not induce SG formation in ZIKV-infected cells. To our surprise, unlike ZIKV E protein, NS1 expression kinetics seemed to be largely unaffected by Regorafenib. Furthermore, translational inhibition by cycloheximide (CHX) treatment affected E protein levels but not NS1 levels in Regorafenib-treated cells. We also ruled out the contribution of proteasomal degradation to the observed effects of Regorafenib treatment as MG132 treatment did not result in accumulation of either E or NS1 proteins; this is consistent with previously published data for ZIKV NS1 and capsid proteins (60, 61). At first glance, these results are counter-intuitive given that both proteins are derived from a single polyprotein. However, our data indicate that Regorafenib treatment leads to inefficient secretion of NS1 resulting in its cytoplasmic retention. It is tempting to speculate that the Regorafenib-induced ER fragmentation is responsible for this retention; possibly by affecting the formation of high-order NS1 oligomers or by disrupting the secretory pathways. This altered morphology also correlates with E protein depletion and limited viral egress, as evidenced by the accumulation of cell-associated viral RNA and reduction in extracellular viral RNA. These combined effects likely contribute to the antiviral activity of Regorafenib on ZIKV; however, a detailed mechanism of action remains unclear and warrants further investigation.

Taken together, this study has identified Dabrafenib and Regorafenib as potent inhibitors of ZIKV at distinct stages of infection despite overlapping host targets. Both inhibitors result in substantial reduction in viral titers at post-entry steps using clinically relevant and tolerable concentrations (62, 63), highlighting their therapeutic potential that is not dependent on prophylactic administration. An added benefit of Regorafenib is its negative impact on NS1 secretion given the established role secreted NS1 plays in the pathogenesis and disease progression of several flavivirus infections (64–66). However, given the potential for strain-specific differences, the efficacy of these inhibitors on additional ZIKV isolates should be examined for broader therapeutic application. Target validation through an orthogonal approach combining pharmacological inhibition and complementation with kinase-dead mutants would help identify the kinases responsible for the observed phenotypes and open new avenues for targeting these specific virus–host interactions whilst sparing host functions. Our findings provide further insight into the biology of ZIKV and have implications for the development of novel antiviral strategies against ZIKV and possibly other flaviviruses.

## MATERIALS AND METHODS

### Cells

A549 cells (human lung ATII adenocarcinoma; ATCC CCL-185) were maintained in Ham’s F-12K Nutrient Mixture (Gibco) supplemented with 10% heat-inactivated fetal bovine serum (FBS; Gibco), 1% penicillin-streptomycin (P/S; Gibco), and 1% GlutaMAX (Gibco). Vero (ATCC CCL-81), Vero E6 (ATCC CRL-1586), and BHK-21 (ATCC CCL-10) cells were maintained in Eagle’s minimum essential medium (EMEM; Sigma-Aldrich) supplemented with 10% FBS, 1% P/S, 20 mM HEPES (Gibco), and 1% GlutaMAX. These cell lines were kept at 37°C and 5% CO_2_. C6/36 cells (kindly provided by Stefanie Becker, University of Veterinary Medicine, Hannover, Germany) were maintained in Leibovitz’s L-15 medium (Gibco) supplemented with 10% FBS, 10% tryptose phosphate broth (Gibco), 1% P/S, 20 mM HEPES, and 1% GlutaMAX, and kept at 28°C in the absence of CO_2_.

### Viruses

ZIKV strain FB-GWUH-2016 (kindly provided by Gülsah Gabriel, Leibniz Institute of Virology, Hamburg, Germany), which was isolated from a human fetal brain with substantial abnormalities (29), was propagated in Vero E6 cells and titrated by plaque assay in Vero cells. DENV2 strain D2Y98P (kindly provided by Sylvie Alonso, National University of Singapore, Singapore), originally isolated from a dengue patient in Singapore in 1998 and passaged about 20 times in C6/36 cells (67), was propagated in C6/36 cells and titrated by plaque assay in BHK-21 cells.

### Compounds

SMKIs [10 mM in dimethyl sulfoxide (DMSO)] were purchased from Selleckchem (Houston, TX, USA), aliquoted, and stored at −20°C. DMSO was purchased from Carl Roth (Karlsruhe, Germany). Dithiothreitol (DTT) was sourced from Invitrogen (Waltham, MA, USA). Cycloheximide was purchased from Sigma-Aldrich (St. Louis, MO, USA).

### Cellular cytotoxicity assay

Cells were seeded at a density of 10^4^ cells/well into opaque-walled flat-bottom 96-well plates (Corning) and incubated the following day with serial twofold dilutions of each SMKI (starting dilution: 10 µM) or DMSO (0.1%; vehicle control) in infection medium (i.e., growth medium containing 2% FBS) for 72 h. Relative cell viability was subsequently assessed using the CellTiter-Glo 2.0 cell viability assay (Promega) according to the manufacturer’s instructions. Control wells containing medium without cells were included to determine background luminescence.

### Virus infections

Cells were seeded at a density of 5 × 10^4^ or 2 × 10^5^ cells/well into flat-bottom 48-well or 12-well plates (both Corning), respectively, the day before the experiment. Medium was aspirated and the cells were inoculated with virus at the indicated MOI [in PFU per cell] or mock-infected for 1 h at 37°C. Afterward, the inoculum was removed, the cells were washed once with DPBS, then replenished with infection medium containing the indicated concentrations of SMKIs or DMSO (vehicle control) and further incubated for the indicated durations.

### Virus titrations

Infectious virus in culture supernatants was titrated by endpoint dilution assay in Vero cells. Briefly, Vero cells were seeded into flat-bottom 96-well plates at a density of 10^4^ cells per well. The following day, the cells were inoculated in quadruplicates with tenfold serially diluted viral suspensions (100 µL per well) and incubated for 4 days at 37°C. Wells were eventually scored for cytopathic effect (CPE) using a light microscope and median tissue culture infectious dose (TCID_50_) values were calculated using the Reed–Muench method (68).

### Immunofluorescence staining

Cells were fixed with 4% paraformaldehyde (PFA) in phosphate-buffered saline (PBS) for 15 min at room temperature (RT), permeabilized with 0.5% Triton X-100 (Sigma-Aldrich) in PBS for 10 min at RT and then blocked with 2.5% normal horse serum (NHS; Cytiva) in PBS for 30 min at RT. Cells were stained with primary antibodies diluted in 2.5% NHS/PBS for 1 h at RT. The following primary antibodies were used: mouse monoclonal anti-flavivirus group antigen-antibody (1:1,000; clone D1-4G2-4-15, EMD Millipore), mouse monoclonal anti-dsRNA antibody (1:500; clone J2, Scicons), chimeric rabbit monoclonal anti-flavivirus group antigen-antibody (1:1,000; clone D1-4G2-4-15, antibodies-online), rabbit polyclonal anti-G3BP1 antibody (1:500; Proteintech), and rabbit polyclonal anti-calnexin antibody (1:500; Sigma-Aldrich). Cells were washed three times with PBS and then incubated with Alexa Fluor 488-conjugated donkey anti-mouse IgG antibody (1:500; Invitrogen) and Alexa Fluor 594-conjugated donkey anti-rabbit IgG antibody (1:500; Invitrogen) along with NucBlue Live ReadyProbes Reagent (Hoechst 33342, Invitrogen) for 1 h at RT in the dark. Images were captured using a Leica DMi8 microscope coupled to a Leica DFC3000 G camera and processed using Leica Application Suite X (LAS X; all Leica Microsystems).

### Western blot analysis

Cells were rinsed once with cold DPBS and then harvested on ice in lysis buffer [50 mM Tris, 150 mM NaCl, 1% Triton X-100, 0.5% sodium deoxycholate, 0.1% sodium dodecyl sulfate (SDS), pH 8.0] containing 1× Halt protease and phosphatase inhibitor cocktail (Thermo Fisher Scientific). Protein extracts were resolved by electrophoresis in 10% or 12% SDS–polyacrylamide gels under reducing conditions, and subsequently transferred onto polyvinylidene difluoride (PVDF) Hybond-P membranes (Amersham). Membranes were blocked with 5% skimmed milk in Tris-buffered saline containing 0.1% Tween-20 (TBST) for 1 h at RT. Membranes were subsequently probed with primary antibodies diluted in 5% bovine serum albumin (BSA)/TBST overnight at 4°C. The following primary antibodies were used: mouse monoclonal anti-ZIKV NS1 antibody (1:1,000; clone GT5212, GeneTex), rabbit polyclonal anti-ZIKV E protein antibody (1:1,000; Biorbyt), rabbit polyclonal anti-phospho-eIF2α (Ser51) antibody (1:2,000; Proteintech), rabbit polyclonal anti-eIF2α antibody (1:2,000; Proteintech), rabbit monoclonal anti-GAPDH antibody (1:2,000; clone D16H11, Cell Signaling Technology), and rabbit monoclonal anti-vinculin antibody (1:5,000; clone 3M13, Sigma-Aldrich). Membranes were washed three times with TBST and then incubated with horseradish peroxidase (HRP)-conjugated goat anti-mouse IgG antibody (1:5,000; Invitrogen) or HRP-conjugated goat anti-rabbit IgG antibody (1:5,000; Abcam) diluted in 5% skimmed milk/TBST for 1 h at RT. After three washes with TBST, blots were developed using SuperSignal West Pico PLUS chemiluminescent substrate (Thermo Fisher Scientific) and subsequently imaged using a ChemiDoc MP imaging system (Bio-Rad). Densitometric analyses were performed using ImageJ (National Institutes of Health). Black dotted lines separating lanes in some immunoblots indicate the removal of portions of the blots for clarity.

### TCA precipitation

Secreted NS1 was concentrated by precipitation with trichloroacetic acid (TCA). To this end, culture supernatants were clarified by centrifugation at 1,000 × *g* for 5 min at 4°C, subsequently transferred to a new reaction tube and centrifuged at 17,000 × *g* for 2 h at 4°C. Nine parts of supernatant were then mixed with one part of 0.15% sodium deoxycholate and incubated for 10 min on ice. Next, TCA (Sigma-Aldrich) was added to a final concentration of 10% and the mixtures were incubated for 20 min on ice, followed by centrifugation at 7,000 × *g* for 20 min at 4°C. Pellets were washed three times with ice-cold acetone and centrifuged at 7,000 × *g* for 5 min. Pellets were air-dried and then dissolved in 100 µL of lysis buffer containing 1× Halt protease and phosphatase inhibitor cocktail. Samples were analyzed by Western blotting as described above.

### RT-qPCR

#### Primer design

Primers and probes were designed based on the nucleotide sequence of an infectious cDNA clone of ZIKV strain Natal RGN (pZV-KU527068-IC; unpublished) and modified to match that of ZIKV strain FB-GWUH-2016 (GenBank accession no. KU870645); oligonucleotide sequences are listed in Tables 2 and 3 . Primers used for reverse transcription contained unique, non-viral tag sequences at the 5′ end of viral strand-specific sequences to allow discrimination between positive- and negative-strand RNA. A primer/probe set specific for the housekeeping gene *GAPDH* was adapted from reference (69). All primer and probe sequences were checked for potential dimer formation using the Multiple Primer Analyzer webtool (Thermo Fisher Scientific). Oligonucleotides were synthesized by Microsynth (Balgach, Switzerland).

**TABLE 2:** Primers used for cDNA synthesis^*a*^

Name	Sequence (5′ to 3′)
RT_ZIKVpos-Tag-3115-RV	AGGAGTATGTCGTCGCATTTGTGTCATTCTTCTCACTCTCAATCCAG
RT_ZIKVneg-Tag-2497-FW	CTCTGGCTACACTGTGATGTGGGTGCTCGGTGGACTTCTC
RT_GAPDH-RV	GAAGATGGTGATGGGATTTC

^*a*^ Unique, non-viral tag sequences are underlined.

**TABLE 3:** Primers and probes used in strand-specific qPCR

Name	Sequence (5′ to 3′)	Conc. (nM)
ZIKVpos-3021-FW	GAGTGTGATCCAGCCGTTA	600
ZIKVpos-Tag-RV	AGGAGTATGTCGTCGCATTT	600
ZIKVpos-probe	FAM-GATCACTGTGTACAGCCTCCTTYCC-BHQ-1	300
ZIKVneg-Tag-FW	CTCTGGCTACACTGTGATGTG	800
ZIKVneg-2604-RV	GGGAGTCAGGATGGTACTTG	800
ZIKVneg-probe	FAM-TCAAAGAAGGAGACGAGATGCG-BHQ-1	300
GAPDH-FW	GAAGGTGAAGGTCGGAGTC	150
GAPDH-RV	GAAGATGGTGATGGGATTTC	150
GAPDH-probe	HEX-CAAGCTTCCCGTTCTCAGCC-BHQ-1	150

#### RNA extraction

For isolation of total cell-associated RNA (which includes viral RNA associated with cell surfaces as well as intracellular RNA), cells were washed two times with DPBS and then processed using the PureLink RNA Mini Kit (Invitrogen). Extracellular viral RNA was isolated from 140 µL of clarified culture supernatant using the QIAamp Viral RNA Mini kit (Qiagen). After elution, RNA was stored at −80°C.

#### cDNA synthesis

Complementary DNA (cDNA) was synthesized from 20 ng of total cell-associated RNA or 5 µL of extracellular viral RNA using SuperScript III reverse transcriptase (Invitrogen) according to the manufacturer’s instructions in the presence of 200 nM of tagged positive strand-specific or negative strand-specific RT primer and 200 nM of *GAPDH*-specific RT primer for 1 h at 55°C, followed by heat-inactivation for 15 min at 70°C.

#### qPCR

Separate duplex reactions (detecting either positive strand or negative strand together with *GAPDH*) or simplex reactions (positive strand only; for extracellular viral RNA quantification) were carried out using iTaq Universal Probes Supermix (Bio-Rad), 10% of the cDNA volume, and primer/probe concentrations listed in Table 3. All reactions were performed on a LightCycler 96 system (Roche) using the following thermal profile: 3 min at 95°C followed by 45 cycles of 15 s at 95°C, 1 min at 60°C, and plate read. Copy numbers were calculated using standard curves generated from gel-purified PCR products of the target regions amplified from pZV-KU527068-IC. To account for differences in cell numbers, cell-associated viral RNA was normalized to *GAPDH* expression levels using a modified 2^−ΔΔCt^ method (69), whereas extra-cellular viral RNA was normalized to equal input RNA volume in the RT-reaction.

### Time-of-addition assays

A549 cells were seeded at a density of 5 × 10^4^ cells/well into flat-bottom 48-well plates the day before the experiment. To determine potential effects on viral entry, the cells were pre-treated with the indicated SMKIs or DMSO for 2 h at 37°C as well as during inoculation with ZIKV (MOI = 3) for 1 h at 37°C (co-treatment). Following viral adsorption, the inoculum was removed, the cells were washed once with DPBS, and then replenished with infection medium without inhibitors. At 18 hpi, the cells were fixed and stained for ZIKV E protein by immunofluorescence, as described above. The percentage of infected cells was determined manually using the Cell Counter plugin in ImageJ.

To study the effects on post-entry steps, the cells were inoculated with ZIKV at an MOI of 3 for 1 h at 37°C, and subsequently replenished with infection medium containing the indicated concentrations of SMKIs or DMSO. At 24, 48, and 72 hpi, culture supernatants were collected and titrated, as described above; additionally, the cells were fixed and stained for ZIKV E protein by immunofluorescence, as described above.

### Cycloheximide–chase assay

A549 cells were seeded at a density of 2 × 10^5^ cells/well and inoculated with ZIKV at an MOI of 3 or mock-infected for 1 h at 37°C. Afterward, the inoculum was removed, the cells were washed once with DPBS, and then replenished with infection medium containing the indicated concentrations of SMKIs or DMSO. At 48 hpi, the cells were exposed to cycloheximide (10 µg/mL), chased for 0, 12, and 24 h, and processed for Western blot analysis as described above.

### Statistical analysis

Comparisons between experimental conditions were made using Mann–Whitney *U* test, two-way ANOVA, Student’s *t* test for unpaired samples or multiple *t* test with Holm–Šidák correction, as specified in the figure legends. *P* values < 0.05 were considered statistically significant. All statistical analyses were performed using Prism (GraphPad).

## Data Availability

All data have been included in the article and supplemental material. All reported data and any additional information required to reanalyze the data are available from the lead contact upon reasonable request.
